# Stimuli-Responsive and Antibacterial Cellulose-Chitosan Hydrogels Containing Polydiacetylene Nanosheets

**DOI:** 10.3390/polym15051062

**Published:** 2023-02-21

**Authors:** Edwin Shigwenya Madivoli, Justine Veronique Schwarte, Patrick Gachoki Kareru, Anthony Ngure Gachanja, Katharina M. Fromm

**Affiliations:** 1 Department of Chemistry, Jomo Kenyatta University of Agriculture and Technology, Nairobi P.O. Box 62000-00200, Kenya; 2 Department of Chemistry, University of Fribourg, 1700 Fribourg, Switzerland

**Keywords:** stimuli responsive, hydrogels, slow release, antimicrobial activity

## Abstract

Herein, we report a stimuli-responsive hydrogel with inhibitory activity against *Escherichia coli* prepared by chemical crosslinking of carboxymethyl chitosan (CMCs) and hydroxyethyl cellulose (HEC). The hydrogels were prepared by esterification of chitosan (Cs) with monochloroacetic acid to produce CMCs which were then chemically crosslinked to HEC using citric acid as the crosslinking agent. To impart a stimuli responsiveness property to the hydrogels, polydiacetylene-zinc oxide (PDA-ZnO) nanosheets were synthesized in situ during the crosslinking reaction followed by photopolymerization of the resultant composite. To achieve this, ZnO was anchored on carboxylic groups in 10,12-pentacosadiynoic acid (PCDA) layers to restrict the movement of the alkyl portion of PCDA during crosslinking CMCs and HEC hydrogels. This was followed by irradiating the composite with UV radiation to photopolymerize the PCDA to PDA within the hydrogel matrix so as to impart thermal and pH responsiveness to the hydrogel. From the results obtained, the prepared hydrogel had a pH-dependent swelling capacity as it absorbed more water in acidic media as compared to basic media. The incorporation of PDA-ZnO resulted in a thermochromic composite responsive to pH evidenced by a visible colour transition from pale purple to pale pink. Upon swelling, PDA-ZnO-CMCs-HEC hydrogels had significant inhibitory activity against *E. coli* attributed to the slow release of the ZnO nanoparticles as compared to CMCs-HEC hydrogels. In conclusion, the developed hydrogel was found to have stimuli-responsive properties and inhibitory activity against E. coli attributed to zinc nanoparticles.

## 1. Introduction

Biosensors play an important role in clinical diagnostics, point-of-care testing, personalized medicine, and pharmaceutical research [1]. In this area, highly sensitive detection systems that have excellent specificity are required for their ability to provide useful insights into an individual’s health [2]. Over the last decade, the scientific community has focused much of its research on biosensor systems applicable to point-of-care (POC) diagnostic devices which can rapidly assess the cause of various illnesses among patients [1,2,3,4,5]. These diagnostic devices facilitate faster and more accurate identification of diseases, which leads to better treatment of patients [1,2]. Among these devices, hydrogels are attracting attention due to their versatility in chemical modification which makes them responsive to external stimuli such as pH [6,7,8,9], or temperature [1,10] and the prospect of encapsulating therapeutic agents such as antimicrobials, e.g., zinc and silver, containing nanoparticles within their matrix [2]. They are developed from both synthetic polymers and biopolymers, and are utilized in tissue engineering, artificial biomedical scaffolds, soft actuators, wound dressings and environmental remediation [6,11]. Chitosan [12,13], cellulose [14,15,16], pectin [17], alginate [18,19], and carrageenan [20,21] are the most attractive surrogates to petroleum-based starting materials due to their relative abundance and availability. Moreover, their biodegradability, biocompatibility, ease of functionalization and gelling properties make them ideal starting materials for developing hydrogels [22,23]. These features can also be enhanced through chemical and physical crosslinking of two different hydrogels to create dual crosslinked polymers [24,25]. In addition, chitosan is a polyelectrolyte that has mild swelling capacity in acidic media but its functionalization by the introduction of a carboxymethyl group and crosslinking to another high swelling capacity polymer such as hydroxyethyl cellulose will further enhance its properties. Moreover, the incorporation of conjugated polymers and other chemical moieties within their matrices further enhances their functionality as they impart the ability to respond to external stimuli [2,26,27]. Conjugated polymers such as polyaniline, polypyrrole, and polydiacetylene (PDA) enable them to function as electrochemical biosensors since the electrochemical properties of the conjugate systems are associated with visible colorimetric changes [26,28]. PDAs are especially attractive as they exhibit a blue to red color transition visible to the naked eye when they are subjected to external stimuli such as changes in temperature, pH, bacterial cells, and aromatic compounds [7,29]. They have also been used in the development of highly sensitive colorimetric probes for the detection of cholesterol [5], ammonia [30], glucose [31], microorganisms [7,32], volatile organic compounds [33], active pharmaceutical excipients, and small and large biomolecules among other compounds [28]. In this study, a hydrogel prepared from polydiacetylene-zinc oxide-carboxymethyl chitosan-hydroxyethyl cellulose (PDA-ZnO-CMCs-HEC) was evaluated for its pH responsiveness, colorimetric transitions and inhibitory activity against *E. coli*. First, chitosan (Cs) was esterified with monochloroacetic acid to obtain carboxymethyl chitosan (CMCs) which was subsequently chemically crosslinked with hydroxyethyl cellulose (HEC) using citric acid as the crosslinking agent to obtain CMCs-HEC hydrogels. To enhance the pH responsiveness of the hydrogels, PDA-ZnO was introduced into the CMCs-HEC hydrogel to obtain a colorimetric pH-responsive PDA-ZnO-CMCs-HEC hydrogel. To better understand the properties such as pH responsiveness, thermal profile, crystallinity, functional group changes upon crosslinking and antimicrobial activity of the hydrogels against *E. coli*, the hydrogels were then analyzed using a Fourier transform infrared spectrophotometer (FT-IR), powder X-ray diffraction (XRD), scanning electron microscopy (SEM), thermal gravimeter analyzer (TGA), and UV-Vis techniques as well as by antibacterial assays.

## 2. Materials and Methods

### 2.1. Materials

Chitosan (medium molecular weight: 100,000–300,000 Da), 10,12-pentacosadiynoic acid (97.0%, HPLC grade), and isopropanol (ACS reagent, ≥99.5%), glacial acetic acid (ACS reagent, ≥99.7%), sodium hydroxide (ACS reagent, ≥97.0%, pellets), and ethyl alcohol (95%) were procured from Sigma Aldrich Co. (Buchs, Switzerland).

### 2.2. Synthesis of CMCs-HEC Hydrogels

**Scheme 1:**
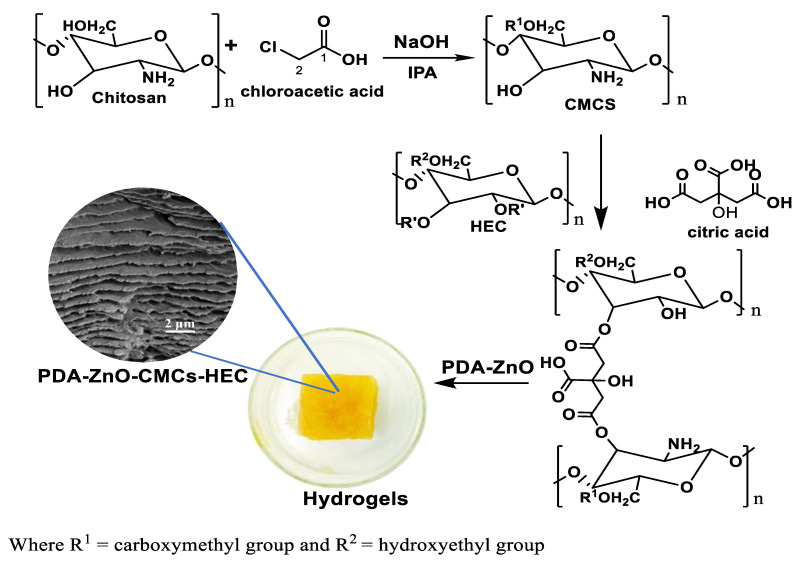
Mechanism of thermochemical crosslinking of CMCs and HEC using citric acid.

Carboxymethyl chitosan (CMCs) was synthesized by esterification using monochloroacetic acid in aqueous media (Scheme 1) [15,34,35].

**Scheme 2:**
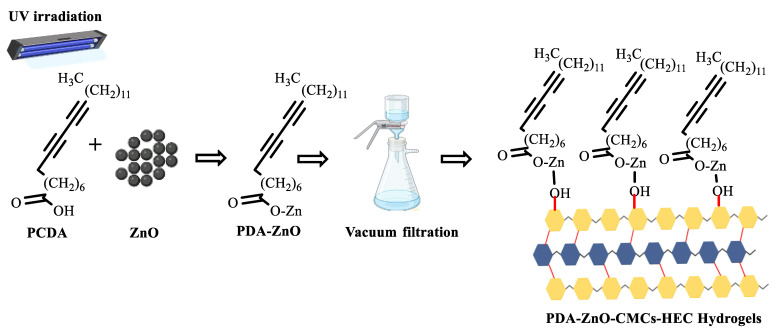
Synthesis of PDA-ZnO-CMCs-HEC.

First, 1 g chitosan (Cs) was dispersed in 20 mL 25 µM NaOH solution followed by the addition of 50 mL isopropyl alcohol and monochloroacetic acid (11–32 mmol/g), respectively. The reaction mixture was heated to 80 °C for 2 h under constant stirring, after which the product was purified by washing repeatedly with 98% ethanol and dried in an oven to constant weight [36]. This was followed by Soxhlet extraction of sodium chloride using methanol as the extracting solvent for 6 h at 70 °C [10,14,37]. To prepare CMCs-HEC hydrogels, different formulations containing HEC and CMCs in the ratios between 1:1–1:5 were prepared in petroleum ether as follows [36]. First, to a series of solutions containing 1 g CMCs in petroleum ether, between 1 and 5 g of HEC were added and stirred for 2 h at room temperature. This was followed by the addition of citric acid (1.04 mmol/g CMCs-HEC) to the solution, which was then stirred for 16 h at 60 °C for the crosslinking to occur, filtered, washed with 98% ethanol and dried to constant weight at 105 °C to obtain CMCs-HEC hydrogels [15,34,35,36,38]. To obtain PDA-ZnO-CMCs-HEC hydrogels, the composite was prepared in situ by crosslinking CMCs and HEC in the presence of 2.67 mmol 10,12-pentacosadiynoic acid containing 2.5 mmol ZnO followed by UV irradiation at 254 nm to form a composite (Scheme 2) [7,30,32].

### 2.3. Swelling Measurements

To evaluate the swelling capacity of the hydrogels as a function of HEC:CMC ratio and pH, 100 mg of the samples were pressed using a laboratory hand press (International Crystal Laboratories, Garfield, NJ, USA) to obtain 7 mm discs. To evaluate the effect of CMC:HEC ratio on the swelling capacity of the hydrogels, the discs were prepared from samples containing HEC-CMC crosslinked in the ratios between 1:1 and 1:5 *g/g*. To study the effect of pH of the swelling media on the swelling capacity, 7 mm disc of HEC:CMC (1:4 *g/g*) were then immersed in ultrapure water whose pH was adjusted to 3, 5, 7, 11, and 14 at 20 °C [27,38,39]. The swelling capacity was then determined in triplicate by removing the discs from the swelling media at 1 min intervals, blotting to remove excess water and recording the weight gained. The degree of swelling was then calculated using the following equation and reported as mean ± SD:

(1) $$\mathrm{Swelling}\;\mathrm{capacity}=\frac{M_{t}-M_{i}}{M_{i}}\;\times \;100$$

where M_i_ and M_t_ are the weights of the hydrogels before and after swelling, respectively [38].

### 2.4. Colorimetric Response to pH Change

The colorimetric response to changes in pH was evaluated by preparing different solutions of PDA-ZnO-CMCs-HEC at 3.3 g/L in distilled water basified with 1 M NaOH to adjust the pH to 7, 8, 10, 12, and 14, respectively. These solutions were then analyzed with a fluorimeter using excitation wavelengths 320 and 365 nm, and measuring from 360 to 620 nm and 400 to 700 nm, respectively. The solutions were then diluted to 400 µL in 2.0 mL of pH-adjusted distilled water and their absorbance was measured using a UV-Vis spectrophotometer from 800 to 200 nm [7,28,29,40].

### 2.5. Characterization of PDA-ZnO-CMCs-HEC

The IR spectra of the PDA-ZnO-CMCs-HEC hydrogels were acquired in the frequency range of 4000–400 cm^−1^ using a Bruker Tensor II FT-IR spectrophotometer model (Bruker, Ettlingen, Germany) [41,42]. UV measurements, fluorescence measurements, and bacterial optical density measurements were measured using Perkin Elmer Lambda 40 UV-Vis Spectrophotometer (Perkin Elmer, Waltham, MA, USA), Perkin Elmer Luminescence spectrometer LS50B (Perkin Elmer, Waltham, MA, USA) and Spark 10 M multimode microplate reader, (Tecan Austria GmbH, Grodig, Austria), respectively. The X-ray diffractograms were obtained using a Bruker D8 Advance Diffractometer (Bruker, Ettlingen, Germany) with a copper tube operating at a voltage and current of 40 kV and 40 mA. The samples were irradiated with a monochromatic CuKα radiation of 0.1542 nm and the diffractograms were acquired between 2θ values of 5°–90° at 0.05° intervals with a measurement time of 1 s per 2θ intervals. The thermal profile of the hydrogels was evaluated using a Mettler Toledo TGA/DSC (Mettler-Toledo GmbH, Greifensee, Switzerland) [41,43]. Approximately 5 mg samples were weighed into 40 µL aluminium crucibles which were then heated from 25–500 °C at 10 °C/min and cooled to 25 °C. Morphological analysis of the composite hydrogels was observed using a Tescan Mira3 LM FE scanning electron microscope (Tescan, Brno-Kohoutovice, Czech Republic) operating under an accelerating voltage of 3 kV. The samples were sputter coated with 4 nm gold before analysis to avoid charging using AGB7340 Agar Sputter Coater (Agar Scientific, Essex, UK) [42,43]. The elemental mapping of the hydrogel was obtained with a Thermo Fischer SEM FEIXL30SFEG equipped with an Oxford Aztec advanced system equipped with an X-MAX 150 mm^2^ Silicon Drift Detector. The EDX mapping was obtained with an accelerating voltage of 15 kV and an acquisition time of 2 h.

### 2.6. Zinc Ion Release Experiments

To determine the concentration of Zn^2+^ ions diffusing from the hydrogels [44], 1 g of the composite containing 0.6 mmol ZnO was immersed in 300 mL distilled water to allow swelling of the hydrogels [45]. Aliquots of 15 mL were subsequently drawn from the solution at 30 min intervals, acidified with 1 mL concentrated nitric acid and the concentration of the solution was measured using a Perkin Elmer ICP-OES Optima7000 DV (Perkin Elmer, Waltham, MA, USA) [46]. 

## 3. Killing Curve Assay

To test the minimum inhibitory concentration (MIC) and minimum bacterial concentrations (MBC), a colony of *Escherichia coli* 25,922 was picked up from a Muller Hinton (MH) agar plate and incubated in 5 mL of MH broth overnight at 37 °C and 180 rpm. Then, a 24 g/L solution of PDA-ZnO-CMCs-HEC in H_2_O was prepared and agitated at 10 rpm overnight [45,46]. A 96 well-plate was filled with different volumes of the hydrogel solutions, and total volumes were completed to 100 uL with MH broth. Bacteria preculture was diluted to 2.0 × 10^4^ cfu/mL MH broth [45,46] after which 100 uL of this culture was inoculated in each well except sterility controls where 100 uL of MH broth was added. The plate was incubated in a humidity cassette at 37 °C and stirred at 72 rpm with absorbance monitoring for 23 h. MIC was then determined as the first concentration for which the absorbance did not increase during the incubation. Then serials of dilutions were performed for all wells, and 100 μL were spread on MH agar plates. They were incubated at 37 °C for 20 h, and the bacterial colonies were counted. MBC was then determined as the first concentration for which no colony grew at all. The same procedure was applied for the negative control [45,46].

## 4. Statistical Analysis

The results obtained in this study were analyzed using Originlab statistical software package (V. 22, OriginLab Corp, Northampton, MA, USA) and reported as their mean ± standard deviations while the least significance difference test (ANOVA, LSD test *p* ≤ 0.05) was used to evaluate the influence of pH and CMC/HEC ratio on the swelling capacity of the hydrogels.

## 5. Results and Discussions

### 5.1. Swelling Behaviour of CMC-HEC Hydrogels

**Figure 1:**
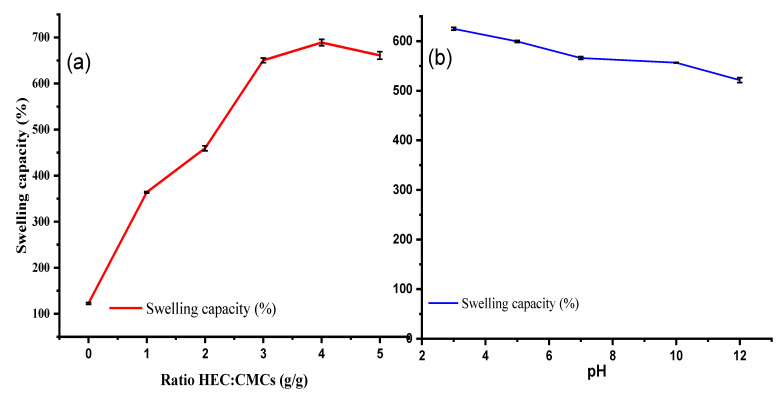
Swelling capacity as a function of (**a**) HEC content and (**b**) pH of the swelling medium.

The swelling capacities of the CMCs-HEC hydrogels (Scheme 1) are depicted in Figure 1.

**Figure 2:**
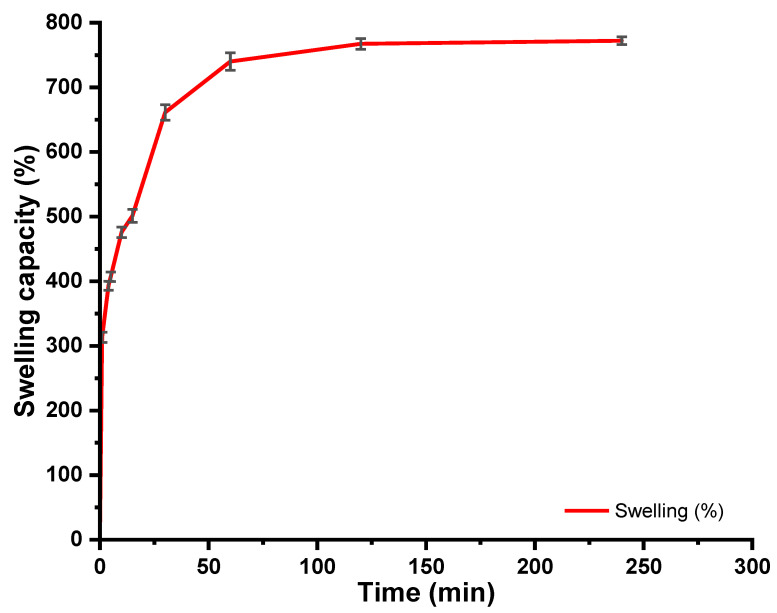
Swelling capacity as a function of time.

As can be observed from Figure 1, while CMCs had a higher swelling capacity as compared to Cs, chemical crosslinking of CMCs with HEC using citric acid resulted in a subsequent increase in the swelling capacity of the resultant hydrogels [36]. Higher swelling capacities were observed when the amount of HEC present in the hydrogels was increased from 0 to 5 g with the highest swelling capacity observed when the ratio of CMCs-HEC was 1:4 *g/g,* respectively. It is worth noting also that maximum water uptake was attained after 20 min in which 1 g of the hydrogel absorbed up to 7 g of water (≈700 %) to attain equilibrium (Figure 2). This fluid uptake is relatively higher as compared to carboxymethyl cellulose (CMC)-hydroxyethyl cellulose (HEC) hydrogels crosslinked with fumaric acid which exhibited a 400% swelling capacity [15,35]. The swelling capacity is dependent on the behaviour of individual components making up the hydrogels, the degree of crosslinking and the type of crosslinker used in the synthesis. For instance, CMC and HEC have been reported to have lower swelling capacities when individually crosslinked with citric acid as compared to when they are crosslinked together [35,36,47,48]. In HEC, the decrease in the swelling capacity was attributed to a lower potential for citric acid adsorption leading to a lower degree of crosslinking brought about by fewer OH groups [36,49,50]. While in CMC the observed lower swelling capacity of 200% was attributed to an increase in electrostatic repulsion between the charged macromolecules of the polyelectrolyte chains that limit water uptake [10,36,51]. As such, the increase in the swelling capacity in CMCs-HEC can be associated with the formation of intermolecular rather than intramolecular crosslinks when HEC is added to the citric acid solution containing CMCs [36]. Moreover, higher swelling capacities are normally observed when the ratio of the constituent polymers favours a higher degree of crosslinks between individual polymers and the crosslinking agent increases hydrogen bond interactions [36,50]. For instance, when crosslinked with divinyl sulfone, CMC and HEC have been reported to have a swelling capacity of 200% as compared to citric acid crosslinked CMC-HEC which had an equilibrium swelling capacity of 900% [52].

The effect of pH on the swelling capacity of the hydrogels was evaluated at pH 3, 5, 7, 10, and 12 (Figure 1b). At low pH values, the swelling capacity of the hydrogels was higher but it gradually decreased as the pH of the swelling media became more basic. The highest swelling capacity of 625% (6g of water per gram of hydrogels) was observed when the pH of the swelling media was 3 while the lowest swelling capacity was observed at pH 12. This phenomenon was attributed to the strong interactions between the two polymers and citric acid, including hydrogen bonding between –COOH and –OH groups and electrostatic interactions between –NH_3_^+^ and –COO^−^ groups. Indeed, as the pH of the swelling media increased, hydrogen bond interactions between –NH_2_ groups and –OH groups and repulsions in the polymer chains resulted in a decline in the swelling capacity. Moreover, it should be noted that using citric acid as the crosslinker leads to a formation of an ester bond (C–O–C) between OH groups present in the two polymers and COOH groups present in citric acid [52]. In the presence of OH^−^ ions, this bond is cleaved forming carboxylic acid salts thereby reducing hydrogen bond interactions between water molecules and the hydrogels. However, at low pH, the protonation of amino groups in chitosan, a water-soluble cationic polyelectrolyte, led to repulsion in the polymer chains thus dissociation of intramolecular hydrogen bond interactions allowing more water uptake into the gel network. Moreover, displacement of Na^+^ present in COONa of carboxymethyl chitosan enhances hydrogen bond formation hence the observed increase in swelling capacity at low pH values. Thus, the ease of solution uptake into the hydrogel at low pH values is attributed to the protonated amine groups in chitosan while the “charge screening effect” of Na^+^ that occurs at higher pH values accounts for the decrease in swelling capacity in a basic medium [38]. In addition, at higher pH values, the electronegative oxygen atom of the water molecule is electrostatically attracted to the positive charge of the metal ions having a larger hydration sphere thereby deswelling the hydrogel. The pH value of normal skin is usually below 5 but once the surface is damaged, the underlying tissue with a pH value of 7.4 is normally exposed. During the early stages of wound healing where the pH is slightly acidic, the PDA-CMC-HEC hydrogels can accelerate cell infiltration and proliferation and facilitate oxygen osmosis thereby aiding wound healing [39,53]. The potential applications of such a hydrogel include releasing antimicrobial agents such as metallic nanoparticles during the initial wound healing phase, which will reduce the extent of inflammation in the inflammation stage and avoid bacterial overgrowth in the fibroblast proliferation phase [54,55,56].

### 5.2. IR Results of CMCs, CMCs-HEC Hydrogels

**Figure 3:**
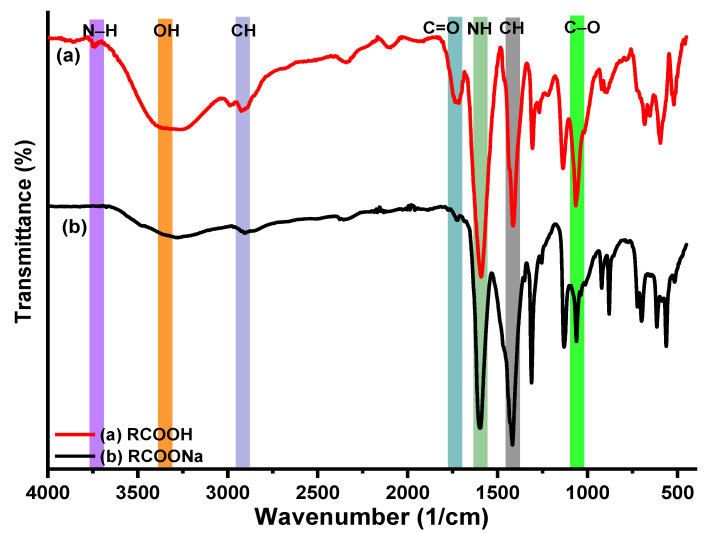
IR spectra of (a) RCOOH, (b) RCOONa.

**Figure 4:**
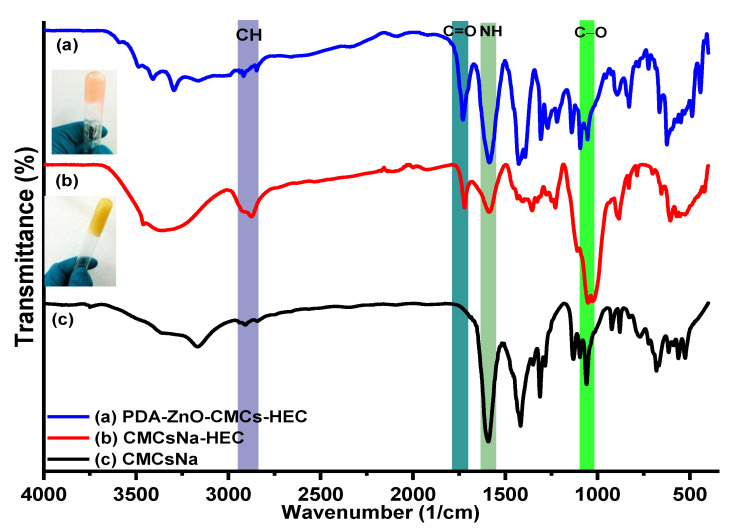
IR spectra of (a) PDA-ZnO-CMCs-HEC, (b) CMCs-HEC, and (c) CMCsNa. Inset CMCs-HEC hydrogels and PDA-ZnO-CMCs-HEC hydrogels.

FTIR spectroscopy was used to follow the introduction of the carboxymethyl group in Cs (Figure 3) and the subsequent crosslinking of HEC and CMCs with citric acid monomers (Figure 4).

The introduction of the carboxymethyl group on CS to form CMCs was manifested by a weak band associated with C=O bonds at 1727 cm^−1^ (Figure 3b). Initially, this band was not observed in both CS and CMCs but ion exchange of CMCs in acetic acid (Figure 3a) resulted in the conversion of RCOONa groups to RCOOH observed by the characteristic C=O vibrational band (Figure 3b) [52]. Characteristic bands of chitosan were observed at 3740 cm^−1^ attributed to NH stretching, OH stretching between 3000–3500 cm^−1^, CH stretching at 2900–2879 cm^−1^, NH bending at 1599 cm^−1^, CH bending at 1401 cm^−1^ and C–O stretching at 1030 cm^−1^ [35,38,47,57]. The band at 1628 cm^−1^ assigned to the NH_2_ group overlapped with the asymmetrical stretching vibration of COONa groups. Treatment of CMCs with acetic acid converted the COONa group to the COOH group evidenced by the formation of a strong characteristic band of C=O at 1738 cm^−1^ present in the free protonated carboxylic groups (Figure S2) [49,57]. Note that, the substitution of carboxymethyl groups on the oxygen in the C_6_ position imparts a pH-dependent anionic character to the molecule [49,52]. From Figure 4, the chemical crosslinking of CMCs with HEC using citric acid as the crosslinking agent led to the formation of a sharp band associated with C=O vibration at 1737 cm^−1^ [57]. This band was attributed to the ester linkage formed when the hydroxyl groups of CMCs and HEC reacted with the carboxyl group of citric acid. On the other hand, the intensity of the NH peak observed at 1599 cm^−1^ decreased as the amount of HEC was increased during the crosslinking reaction (Figure S3) [35,38,52]. PDA nanosheets synthesized in the absence of the hydrogels were also characterized by IR (Figure S4). The vibrational bands originating at IR ν_max_ (cm^−1^): 2918−2847 (C−H stretch), 1690 (C=O stretch), and 722 (C−H bend) were observed [7]. In the presence of CMCs-HEC, a doublet was observed at 2922 cm^−1^ and 2852 cm^−1^ (C−H stretch), while the bands at 1419 cm^−1^ in CS shifted to 1422 cm^−1^ with a shoulder at 1394 cm^−1^.

### 5.3. XRD Results of CMCS-HEC Composite

**Figure 5:**
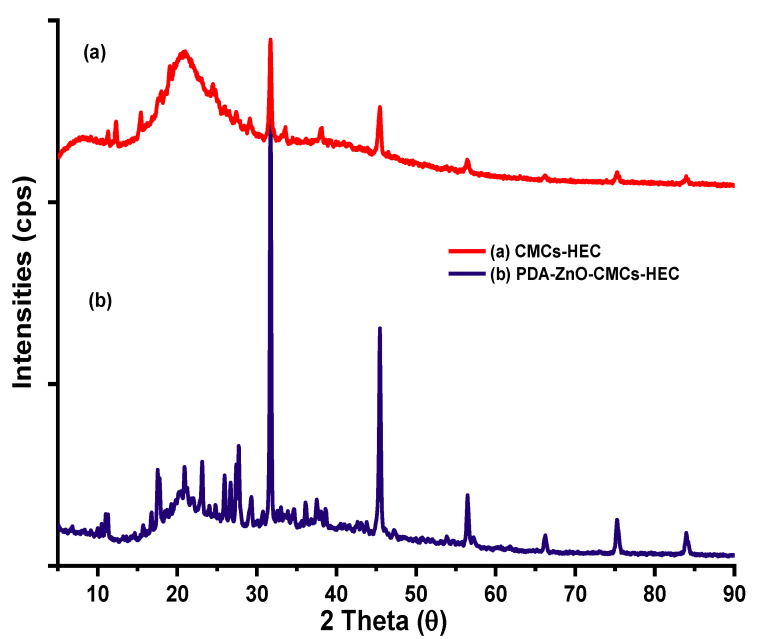
X-ray diffractograms of (a) CMCs-HEC and (b) PDA-ZnO-CMCs-HEC hydrogels.

The X-ray diffractograms of CMCs-HEC and PDA-ZnO-CMCs-HEC hydrogels are depicted in Figure 5.

From Figure 5, it can be observed that there were no remarkable changes in the diffractograms after chemical crosslinking of CMCs and HEC with citric acid. Typically, chitosan exhibits broad peaks centred at about 2θ = 18°and 32° attributed to the presence of OH and NH_2_ groups which form stronger interactions that impart some degree of crystallinity. On the other hand, being a precursor of cellulose, HEC exhibits diffraction peaks that are typically observed in cellulose nanofibers at 2θ angles of 16°, 22°, and° which have been attributed to the diffraction planes of (101) and (002) characteristic of cellulose I (Figure S5) [43,58]. Chemical crosslinking CMCs and HEC with citric acid resulted in one peak centred at 2θ = 21 being observed with sharp peaks at 2θ values of 31°, 37°, 45°, 56°, and 75°. The broad peak was attributed to the overlap of broad peaks of Cs and HEC while the sharp peaks were attributed to citric acid used as a crosslinking agent in the hydrogels (Figure S5). The incorporation of PDA-ZnO within the hydrogels was linked to the sharp peaks observed at 2θ values of 32°, 36°, 48°, 57°, 68°, 75°, and 85° associated with (100), (101), (102), (110), (103), and (113) crystal planes of crystalline ZnO nanoparticles which had an average crystallite size of 18.22 ± 4.7 nm calculated from the Scherrer equation [7,59].

### 5.4. Thermal Profile of Cs, CMCS, and Hydrogels

**Figure 6:**
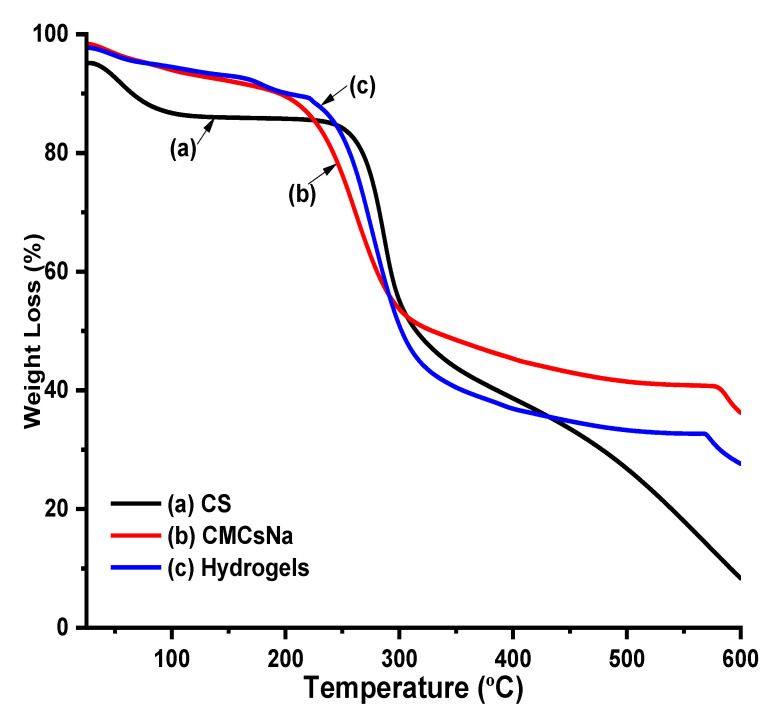
TGA thermograms of (a) Cs, (b) CMCsNa, and (c) hydrogels.

**Figure 7:**
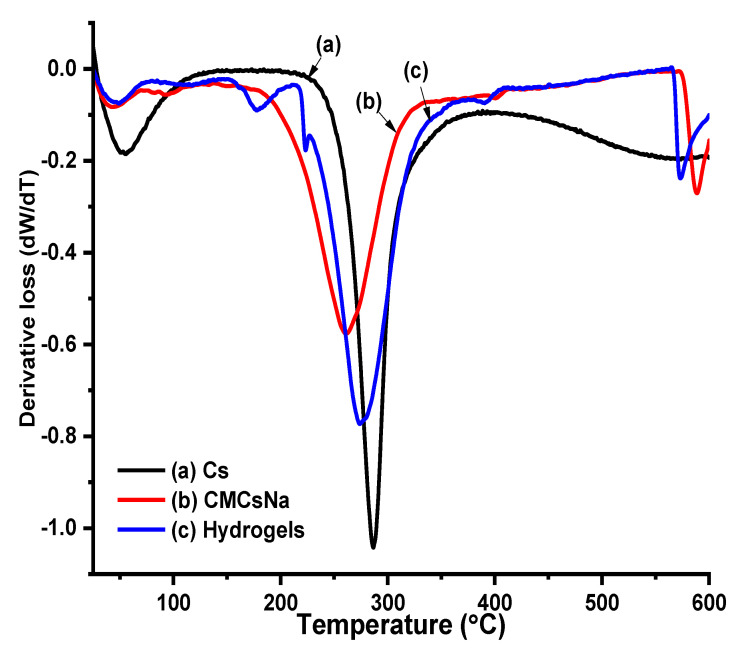
DTGA thermograms of (a) Cs, (b) CMCsNa, and (c) hydrogels.

The thermal profiles of Cs, CMCs, and the prepared hydrogels were investigated using TGA/DTGA and the results are depicted in Figure 6 and Figure 7.

Both CMCs and Cs had two degradation stages in which, the first degradation stage accounted for water loss in CMCs and the hydrogels, which was slower than for Cs particles (Figure 6 and Figure 7). The second and third mass loss in the DTGA thermograms of the hydrogels was observed in the range between 175–300 °C. The onset degradation temperature and ash content for Cs was higher as compared to that of CMCs and the hydrogels. The first degradation stage observed below 100 °C was attributed to the evaporation of water molecules adsorbed on the surface of the Cs, CMCs, and hydrogels [15,35]. For Cs and CMCs, the second degradation stage that is linked to the breaking down of the anhydroglucose units was observed to occur at 286 and 259 °C, respectively [60]. As for the hydrogels, this degradation stage was observed to occur at 273 °C since a second degradation stage associated with the degradation of the crosslinking agent was observed at 175 °C. In this study, the CMCs-HEC hydrogel possessed lower thermal stability than the corresponding precursors as CMCs and HEC have maximum degradation temperatures of 284 and 280 °C, respectively [15,35].

### 5.5. UV-Vis Spectra of Polydiacetylene

**Figure 8:**
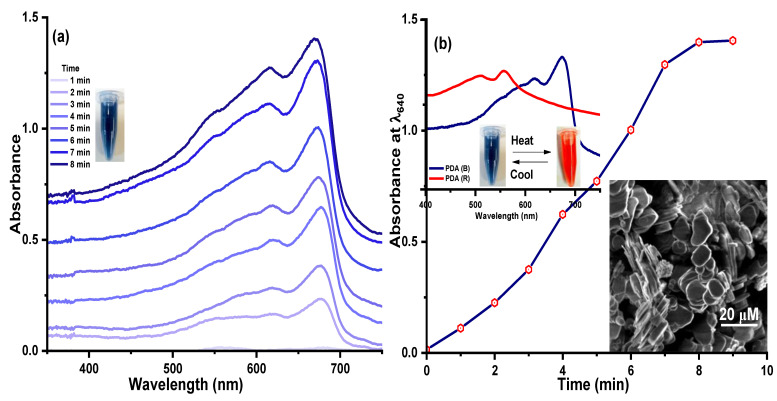
Absorption spectra of (**a**) PDA-ZnO, (**b**) plot of A_670nm_ vs. time. Inset thermochromic shift at 65 °C and PDA nanosheets micrograph.

Figure 8 depicts the UV-Vis spectra of the photopolymerization reaction that converted PDCA to PDA and the subsequent thermochromic shift.

As illustrated in Figure 8, the photopolymerization of 10,12-pentacosadiynoic acid (PCDA) upon irradiation with UV radiation occurred as a function of time. The plot shows a sharp increase in the absorbance at the onset of the photopolymerization which starts to level off after irradiation for 6 min, an indication of the formation of PDA nanosheets (Figure 8b inset). The colour of the solution, which changed from clear colourless to dark blue, was also another indicator of the complete polymerization, but upon exposure of this solution to higher temperatures, it changed to red (Figure 8b inset) [7,33,59,61]. Interaction of the monomers upon radiation resulted in the formation of a peak centred at 650 nm that increased with longer photopolymerization time as the nanosheets are formed (Figure 8a). However, due to the presence of an extended π-electron system composed of alternating carbon multiple bonds, this peak can shift to higher or lower wavelengths when the sheets are exposed to external stimuli such as thermal, chemical, and mechanical. At higher temperatures, however, the hypsochromic shift observed at 550 nm when blue PDA nanosheets change to red PDA nanosheets is a result of the conjugation effect which endows the chromatic properties to the material giving rise to the observed shift in λ_max_ (Figure 8b) [2].

### 5.6. Colorimetric Response of the Hydrogels to pH

**Figure 9:**
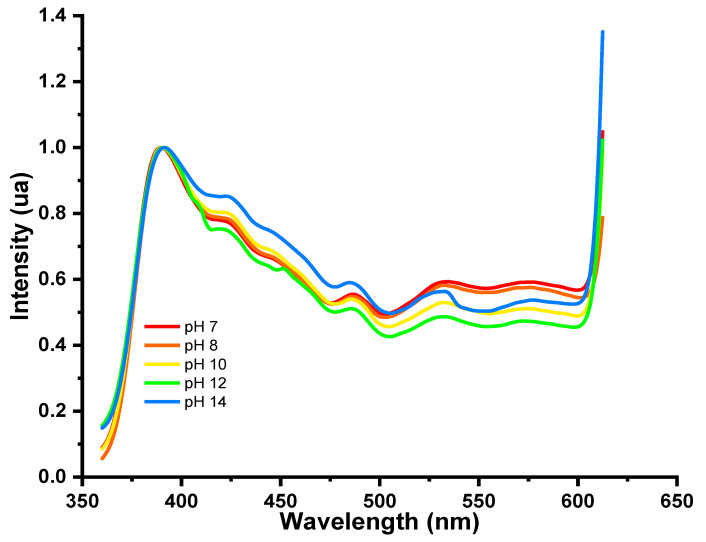
Fluorescence spectra of PDA-ZnO-CMCs-HEC as a function of pH.

**Figure 10:**
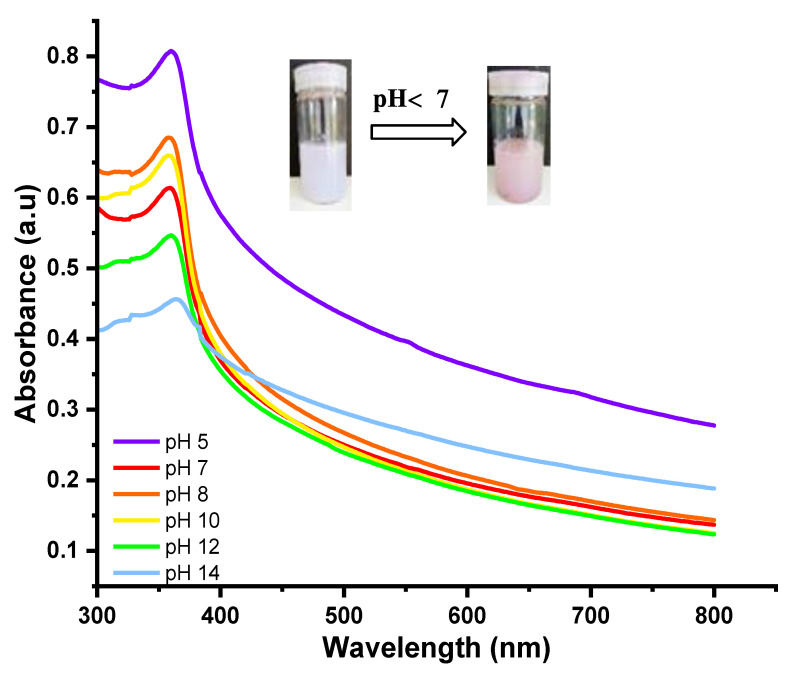
Absorbance spectra of PDA-ZnO-CMCs-HEC as a function of pH. Inset colour transition at higher pH.

Figure 9 and Figure 10 depict the fluorescence and UV spectra of the hydrogel solutions as a function of pH.

From Figure 9, it was observed that PDA-ZnO-CMCs-HEC was only slightly fluorescent, and no major changes were observed when the pH was varied. Nevertheless, the hydrogel depicted negative solvatochromism when in contact with basic media which is illustrated in the UV absorbance spectra (Figure 10). The observed hypsochromic shift upon changes in pH between pH 5 (max at 364 nm) and pH 14 (max at 364 nm) was also visually observed as the hydrogel changed its colour from pale purple to pale pink (Figure 10 inset). This colorimetric response was also evident when PDA-ZnO was exposed to pH changes, though in this case, it resulted in a hypsochromic shift on exposure to basic pH [7,28,29,40]. Similar observations have been made in the literature as various composite materials containing PDA showed colorimetric transition when they were exposed to basic pH [7,28,29,40]. This transition has been reported to be important as it can be used as a means to detect the onset of infection in wounds as, during infection, the pH around the wound area has been reported to be basic [7,28,29,40].

### 5.7. SEM Micrographs

**Figure 11:**
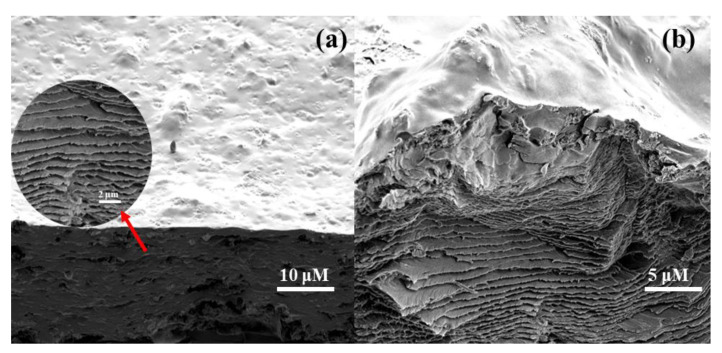
SEM micrographs of hydrogels (**a**) surface and (**b**) cross-section. Inset hydrogel cross-section.

**Figure 12:**
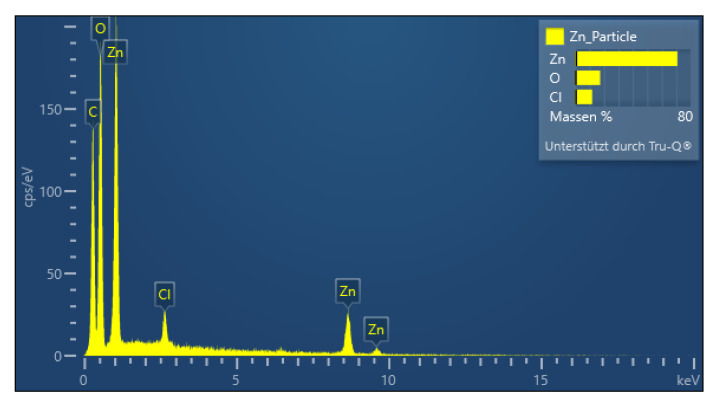
EDS spectra showing the elemental composition of the composite films.

The nanostructures formed during the formation of PDA-ZnO-CMCs-HEC were studied using SEM and the results are depicted in Figure 11 and Figure 12.

In this study, the presence of amine groups and a plethora of OH functional groups in the hydrogel network are instrumental in the structuring and stabilization of the PDA assemblies within the hydrogel network. The nanocomposite comprised a mixture of nanorods, nanosheets and spherical hollow rings which was attributed to the composition making up its matrix (Figures S6–S8) [62]. Before chemical crosslinking, HEC appeared as thick fibres while CMCs appeared as sponge-like amorphous materials (Figure S7) but after chemical crosslinking and in situ synthesis of PDA-ZnO, the composition of the resultant hydrogel changed (Figure S8). ZnO nanoclusters which appeared as rods of different sizes and lengths within the hydrogel network were confirmed by EDS spectra (Figure 12) and elemental mapping of the composite (Figure S9). These nanorods were also observed when the hydrogels were used to prepare composite films through dissolution and solvent casting in PET moulds. In this case, while the surface of the resultant dried hydrogel films was smooth, observation of its cross-section revealed that it comprised multiple layers stacked together in a repeating pattern (Figure 11) similar to multi-layered hydrogels reported in the literature. These highly porous and interconnected multiple layers are a result of strong ionic and electrostatic interactions within the hydrogel network which have been shown to be responsible for the formation of interlayer spaces in a composite hydrogel [63,64]. The formation and growth of each layer are related to the diffusion of the crosslinker with the interlayer spaces fine-tuned by changing the degree of crosslinking between layers [63,64,65]. For the case of PDA-ZnO-CMCs-HEC, the interaction between zinc ions and the hydroxyl and amine functional groups found in CMCs-HEC induced a chelation effect which lead to the formation of multi-layered hydrogels [63,64,65]. In addition, polyanions such as Ca^2+^, Al^3+^, Cu^2+^ and other polyvalent inorganic cations have been used as crosslinkers to prepare multi-layered hydrogels with arbitrary shapes, including onion-like, tubular, and star-like from combinations of different polymers such as alginate, carboxymethyl cellulose, chitosan, and agar, among others [63,64,65].

### 5.8. Kinetics of Zn^2+^ Release from the Nanocomposites

**Figure 13:**
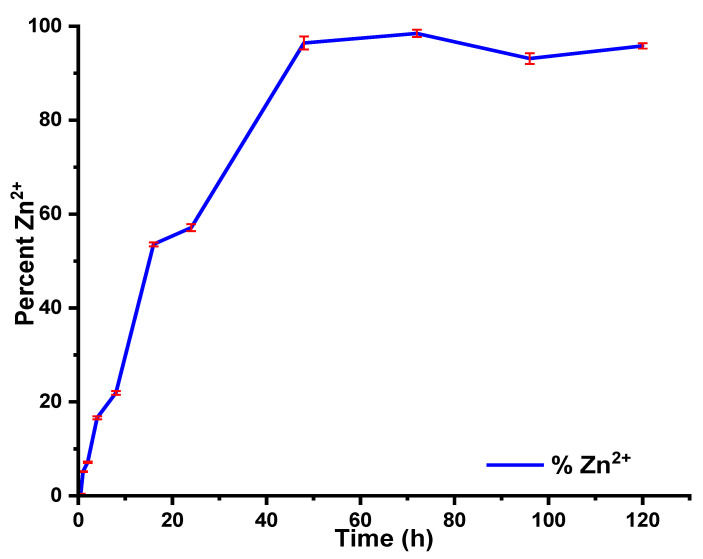
Percent Zn^2+^ ions diffusing from the hydrogels per unit time (h).

Figure 13 depicts the concentration of zinc ions released from the hydrogels over a period of 120 h as determined by ICP-OES measurements.

From Figure 13, it can be observed that the percent concentration of zinc ions released increases over time with the highest amount released being observed after 72 h before it gradually became constant. From the experimental data obtained in this study, it was observed that the amount of zinc ions released was initially low during the first few hours after the hydrogels were immersed in the absorbing media but it gradually increased with time. After 1 h, the concentration of Zn^2+^ ions in solution was found to be 6.74 ppm but it gradually increased to 121 ppm (93% Zn^2+^) after 96 h (Figure S10) remaining constant afterwards [46,66].

Similar observations were made when AgNPs were embedded within chitosan-PEG hydrogels and titanium dioxide nano-capsules in which there was a gradual increase in the concentration of AgNPs being released over several days [46,66]. It is worth mentioning that diffusion of Zn^2+^ ions loaded into a polymer matrix usually implies water penetration into the matrix, hydration, swelling, diffusion of the dissolved substance and or erosion of the gelatinous layer. The amount of encapsulated moiety released from the matrix is dependent on the loading efficiency, the solution pH, and the nature of the encapsulated substance and polymer used [46,66].

### 5.9. Antimicrobial Assays

**Figure 14:**
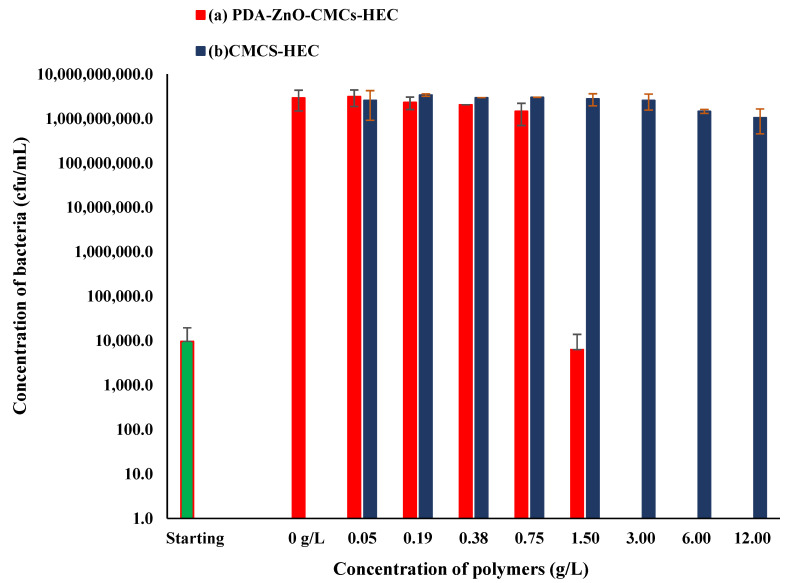
Minimum bacterial concentration (MBC) of alive *E. coli* 25,922 isolates after 23 h in the presence of (a) PDA-ZnO-CMCs-HEC and (b) CMCs-HEC hydrogels.

The inhibitory activity of the hydrogel was evaluated by killing curve assay and the results are depicted in Figure 14.

**Figure 15:**
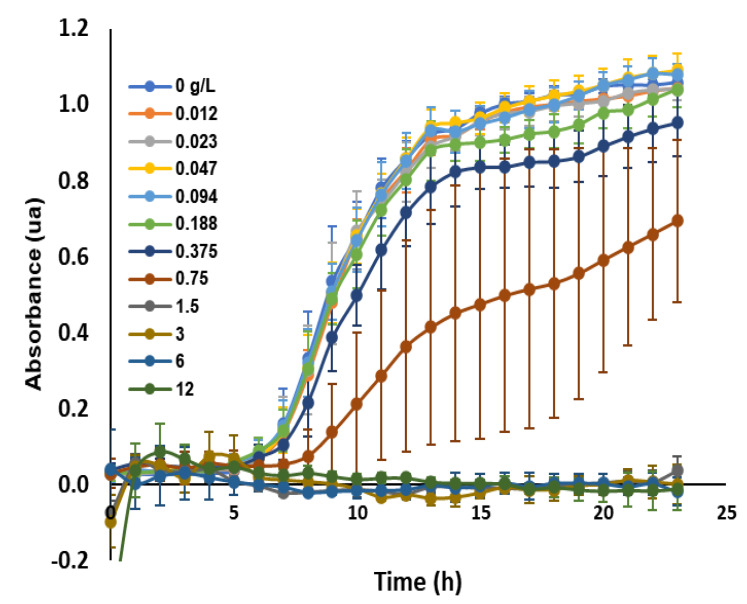
Growing bacteria in the presence of (MIC) of PDA-ZnO-CMCs-HEC.

**Figure 16:**
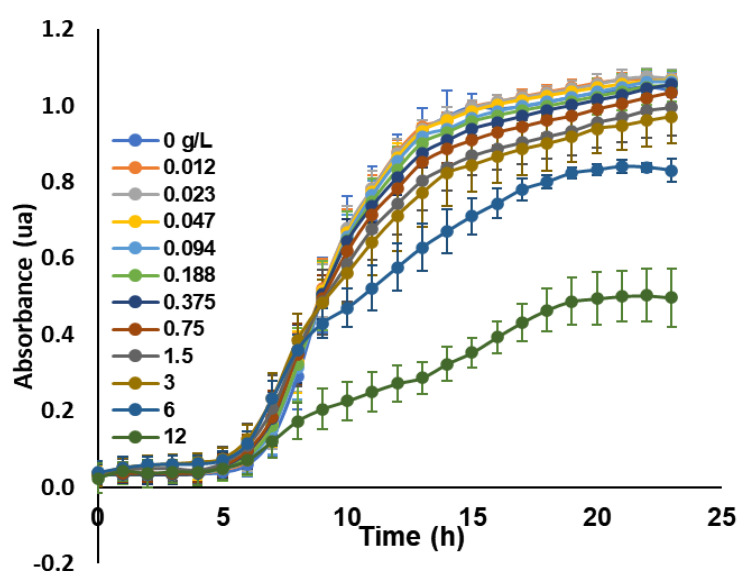
Growing of bacteria in the presence of CMCs-HEC (negative control).

**Figure 17:**
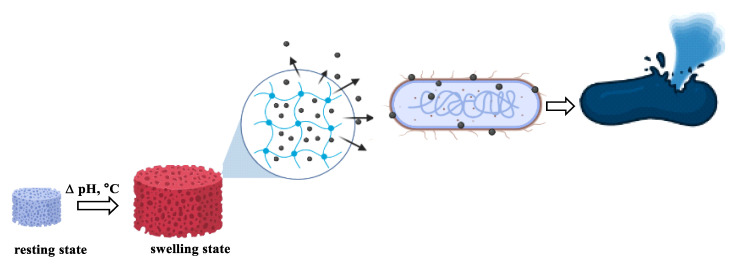
Mechanism of antimicrobial activity of hydrogels against *E. coli*.

As can be observed in Figure 14, the bacterial concentration at the start was 1.0 × 10^−4^ cfu/mL but PDA-ZnO-CMCs-HEC was able to inhibit the growth of *E. coli* as compared to CMCs-HEC that was used as a control (Figure 15 and Figure 16). The composite hydrogels were able to absorb liquid from the culture media which necessitated the flow of Zn^2+^ ions from the hydrogels into the surrounding medium thereby preventing the growth of bacteria [66]. As for the case of CMCs-HEC, the low inhibition activity observed was a result of the absence of ZnO nanoparticles which were present in PDA-ZnO-CMCs-HEC [66]. The PDA-ZnO-CMCs-HEC scaffolds showed excellent antibacterial activity toward clinical *E. coli* compared with CMCs-HEC scaffolds in vivo attributed to the release of Zn^2+^ ions from the composite hydrogels. From kinetic measurements (Figure 13), it was observed that after 24 h, the amount of zinc ion released from the composite hydrogels into swelling media was 74 ppm which was linked to the antibacterial activity being observed during bacterial studies (Figure 14 and Figure 15) as the composite hydrogel without ZnO that did not inhibit bacterial growth (Figure 16). This strain of *E. coli* has an MBC (Figure 14) and an MIC (Figure 15) of 1.5 g/L for PDA-ZnO-CMCs-HEC as compared to CMCs-HEC which did not exhibit any inhibitory activity against the bacteria. It has been shown that the incorporation of metal nanoparticles such as ZnO and AgNPs in a nanocomposite enhances their inhibitory activity thereby preventing the onset of infections. This inhibitory activity is associated with the gradual and sustained release of the antimicrobial ZnO nanoparticles which interact with the bacterial cell causing cell membrane disruption, the infiltration of cell components and, ultimately, bacterial cell lysis (Figure 17) [45,46,66]. Moreover, chitosan alone and chitosan functionalized with cyclohexanone and 2-N-methylpyrrolidone have been shown to have a minimum inhibitory concentration of 50 g/mL for *E. coli* [39].

## 6. Conclusions

We rationally combined PDA-ZnO nanosheets as a stimuli-responsive matrix into CMCs-HEC hydrogel to obtain a pH- and thermal-responsive PDA-ZnO-CMCs-HEC hydrogel with inhibitory activity against *E. coli*. The swelling capacity of the hydrogels was pH dependent as it had a higher swelling capacity in acidic pH as compared to basic pH, though the presence of PDA-ZnO within the hydrogel imparted a colorimetric response triggered by a change in pH from acidic to basic pH. This response resulted in a colour transition from purple to pale pink visible to the naked eye and as such, it is hypothesized that this property can be utilized as a sensing mechanism to indicate the onset of infection in a wound. Moreover, the incorporation of ZnO within the hydrogel ensured that the composite had inhibitory activity against *E. coli* as compared to the composite without nanoparticles.

## Data Availability

The data presented in this study are openly available in Zenodo at 10.5281/zenodo.7500538., reference number 7500539.

## Supplementary Materials

The following supporting information can be downloaded at: https://www.mdpi.com/article/10.3390/polym15051062/s1, Figure S1: IR spectra of (a) chitosan (CS) and (b) carboxymethyl chitosan (RCOOH); Figure S2: IR spectra of hydrogels prepared by crosslinking different ratios of HEC, Figure S3: IR spectra of (a) PDA and (b) PDA-ZnO NPs; Figure S4: X-ray diffractogram of CS, HEC and CMCs-HEC; Figure S5: X-ray diffractogram of citric acid; Figure S6: SEM Micrographs of (a) HEC and (b) CMCsNa; Figure S7: SEM Micrographs of PDA nanosheets on PDA-CMCs-ZnO-HEC; Figure S8: SEM micrographs of ZnO nanorods on PDA-ZnO-CMCs-HEC; Figure S9: Elemental mapping showing (a) carbon (b) oxygen (c) zinc (d) chlorine; Figure S10: Concentration of Zn^2+^ ions diffusing from hydrogels per unit time.
