# Supplementary material for: Stimuli-Responsive and Antibacterial Cellulose-Chitosan Hydrogels Containing Polydiacetylene Nanosheets
Source: Polymers (Basel). 2023 Feb 21;15(5):1062. doi: 10.3390/polym15051062 (PMC10005511; doi:10.3390/polym15051062)
Supplement: Supplementary file 1 [file polymers-15-01062-s001.zip › polymers-2207812-supplementary.pdf]

Supplementary file PDA-CMCs-HEC hydrogels

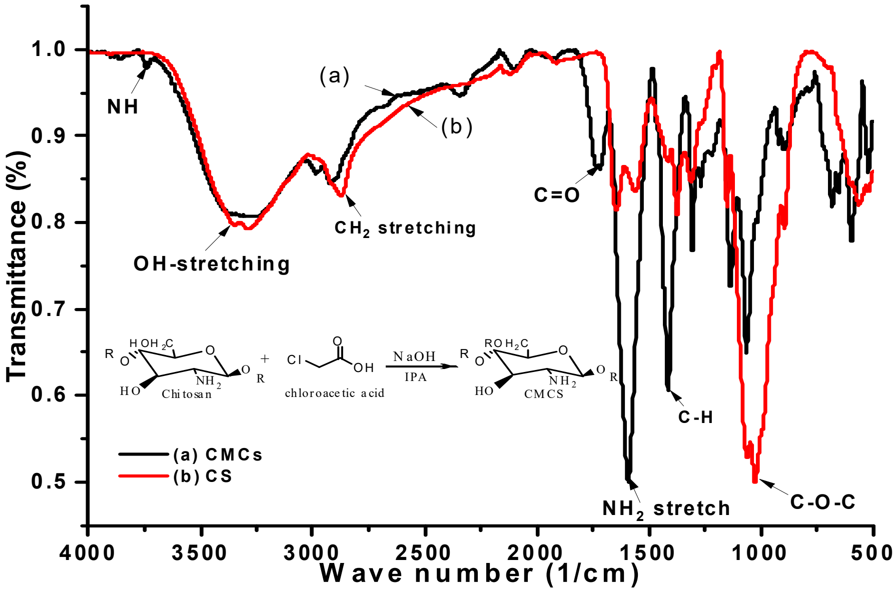

Figure S1: IR spectra of (a) chitosan (CS) and (b) carboxymethyl chitosan (RCOOH)

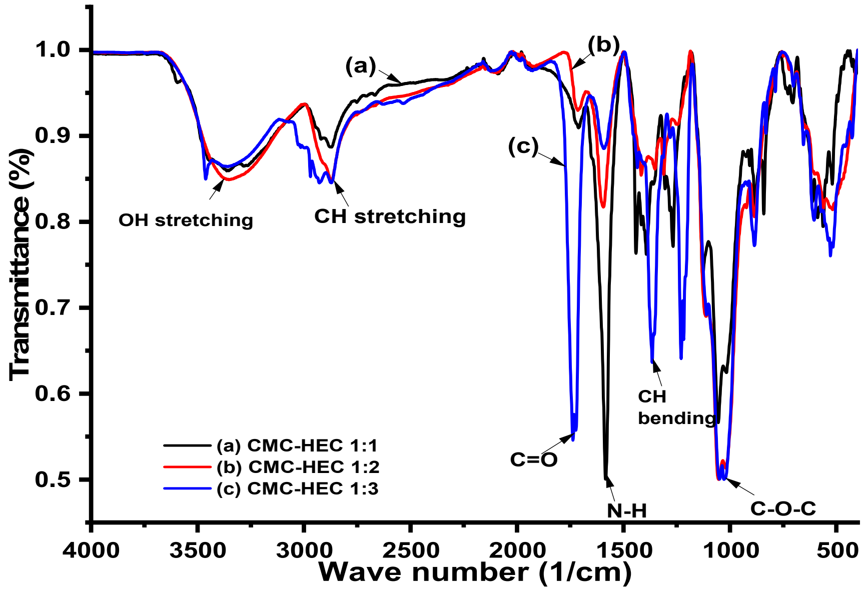

Figure S2: IR spectra of hydrogels prepared by crosslinking different ratios of HEC

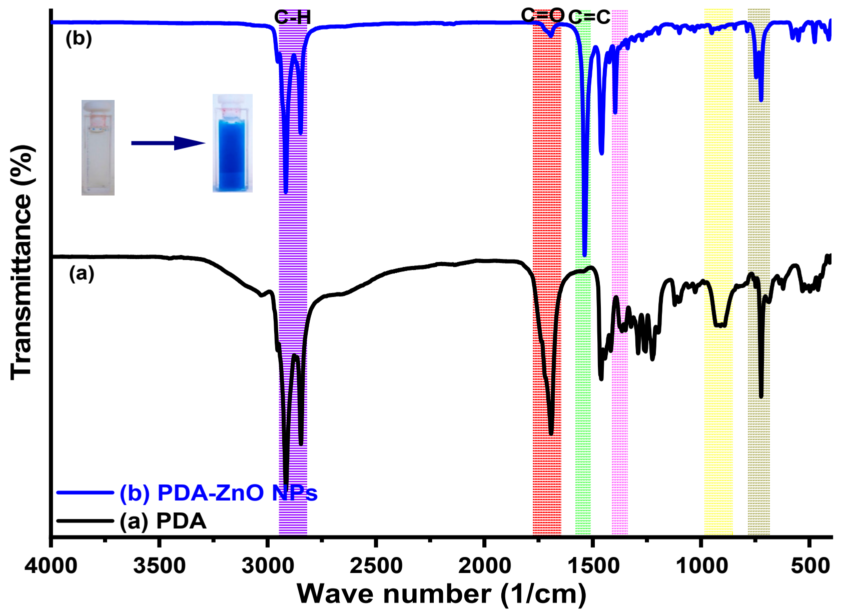

Figure S3: IR spectra of (a) PDA and (b) PDA-ZnO NPs

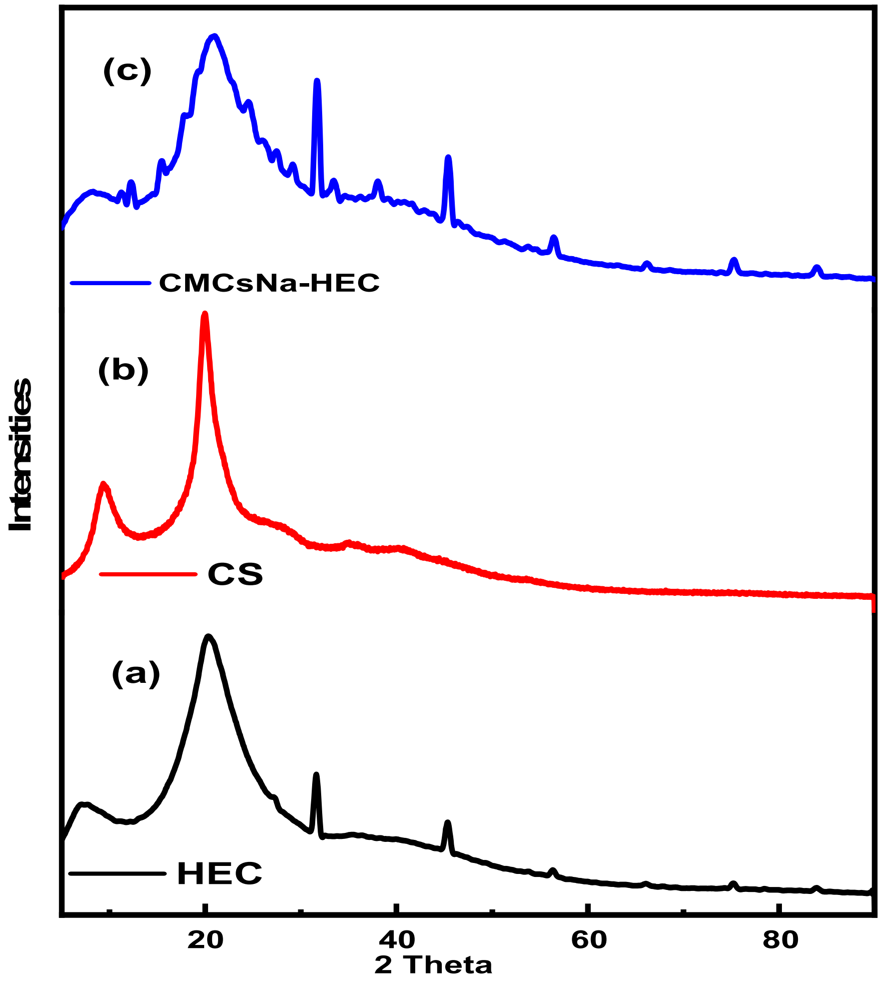

Figure S4: X-ray diffractogram of CS, HEC and CMCs-HEC

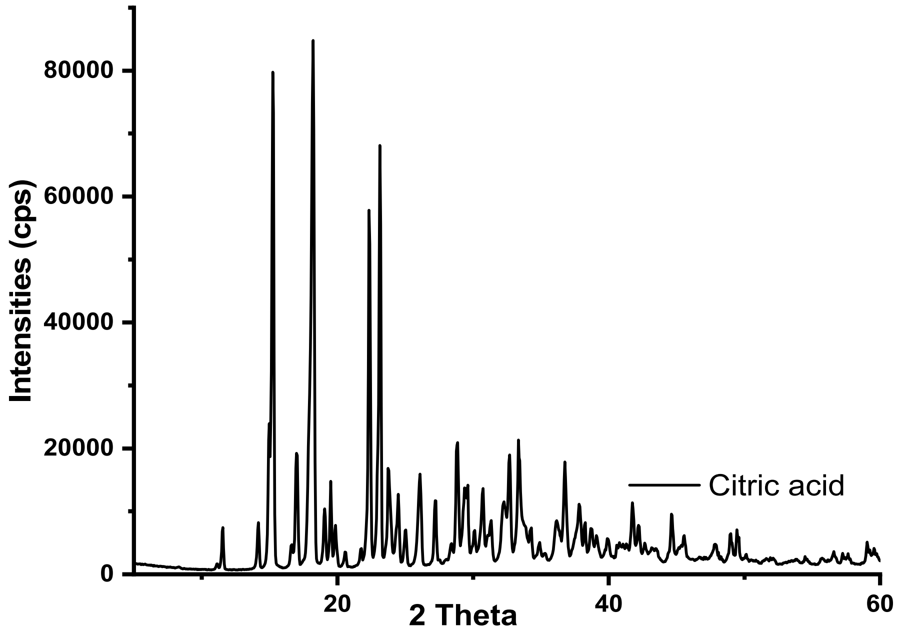

Figure S5: X-ray diffractogram of citric acid

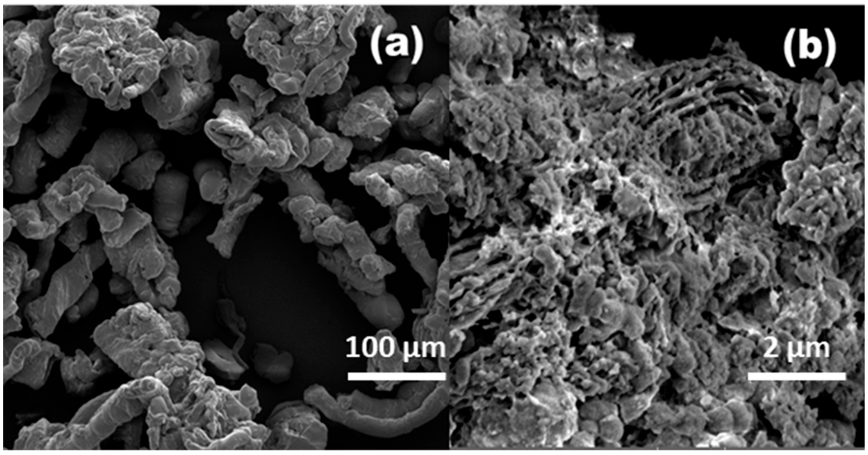

Figure S6: SEM Micrographs of (a) HEC and (b) CMCsNa

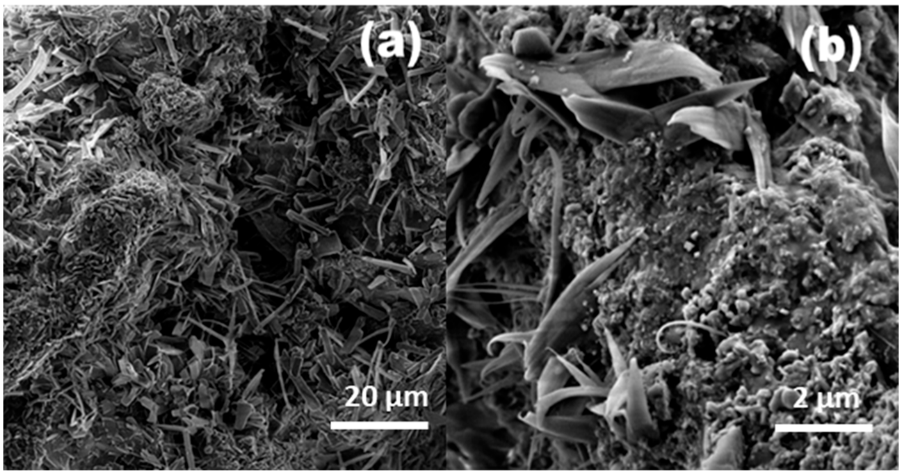

Figure S7: SEM Micrographs of PDA nanosheets on PDA-CMCs-ZnO-HEC

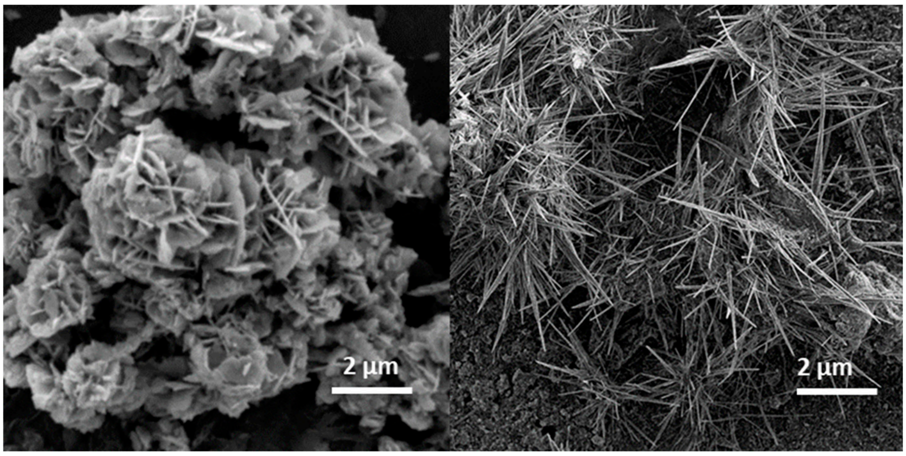

Figure S8: SEM micrographs of ZnO nanorods on PDA-ZnO-CMCs-HEC

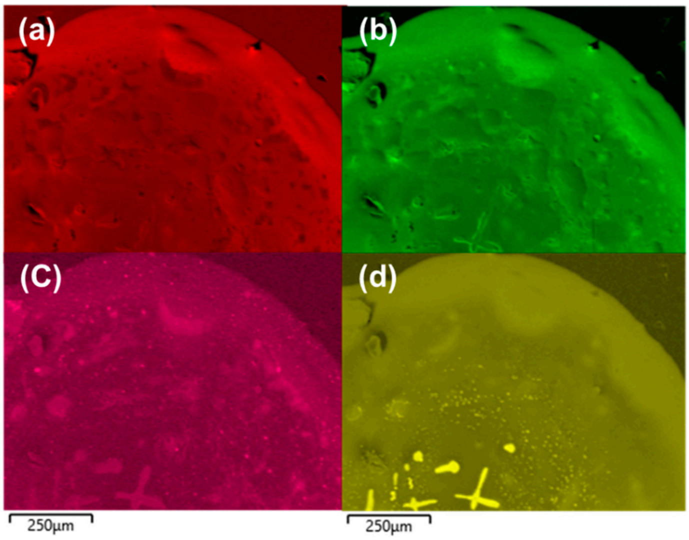

Figure S9: Elemental mapping showing (a) carbon (b) oxygen (c) zinc (d) chlorine

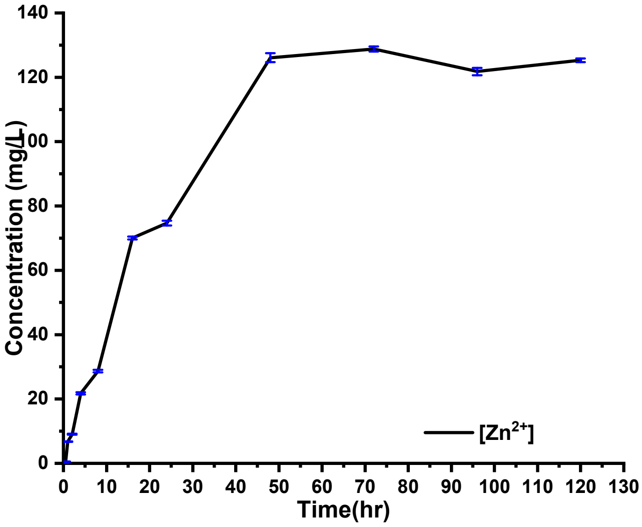

Figure S10: Concentration of Zn^{2+} ions diffusing from the hydrogels per unit time (h)
